# Gender Differences in Child Poverty and Social Exclusion Across Europe: A Comparative Focus on Serbia

**DOI:** 10.3390/children12070854

**Published:** 2025-06-27

**Authors:** Svetlana Vukosavljevic, Snezana Radovanovic, Olgica Mihaljevic, Nebojsa Zdravkovic, Vladislava Stojic, Stefan Milojevic, Jovana Radovanovic, Milos Stepovic, Katarina Janicijevic, Nevena Folic, Marija Radovanovic, Branka Markovic

**Affiliations:** 1 Health Center Dimitrije Pitovic, 31260 Kosjeric, Serbia; svetlanavukosavljevic76@gmail.com; 2 Faculty of Medical Sciences, University of Kragujevac, 34000 Kragujevac, Serbia; stefan.milojevic@fmn.kg.ac.rs; 3 Department of Social Medicine, Faculty of Medical Sciences, University of Kragujevac, 34000 Kragujevac, Serbia; jovanarad@yahoo.com (S.R.); kaja.andreja@yahoo.com (K.J.); 4 Department of Pathophysiology, Faculty of Medical Sciences, University of Kragujevac, 34000 Kragujevac, Serbia; vrndic07@yahoo.com; 5 Department of Medical Statistics and Informatics, Faculty of Medical Sciences, University of Kragujevac, 34000 Kragujevac, Serbia; nzdravkovic@gmail.com (N.Z.); vladislavastojic@gmail.com (V.S.); 6 Faculty of Business Economics, EDUCONS University, 21208 Sremska Kamenica, Serbia; 7 Department of Epidemiology, Faculty of Medical Sciences, University of Kragujevac, 34000 Kragujevac, Serbia; radovanovicjovanaaa@gmail.com; 8 Department of Anatomy, Faculty of Medical Sciences, University of Kragujevac, 34000 Kragujevac, Serbia; 9 Department of Pediatrics, Faculty of Medical Sciences, University of Kragujevac, 34000 Kragujevac, Serbia; nevena.folic@yahoo.com (N.F.); marija9@verat.net (M.R.); 10 Clinic for Pediatry, University Clinical Centre of Kragujevac, 34000 Kragujevac, Serbia; 11 Faculty of Sport and Physical Education, University of Belgrade, 11000 Belgrade, Serbia; branka.markovic@fsfv.bg.ac.rs

**Keywords:** poverty, social exclusion, children, gender inequality, European countries

## Abstract

**Background**: Child poverty is a critical issue that affects millions of children worldwide and represents a global issue. This article focuses on exploring the risk of child poverty and social exclusion across Europe, with Serbia being in a comparable position with respect to other countries with different levels of development. **Methods**: This is an epidemiological, retrospective, and descriptive study based on data on the national populations of the European countries within our research interest. The data analyzed in this study was taken from publicly available datasets from Eurostat. The indicators of interest were the ones considering the social inclusion of children in the time period between 2014 and 2023 for all European countries available in the datasets. **Results**: The indicator of the risk of poverty and social exclusion showed a decreasing trend in the majority of countries; but still, seven countries showed an increase during the observation period of one decade. The highest percentage was in Romania and the lowest in Slovenia. The indicator of the risk of poverty exhibited a significant difference in terms of gender (being mostly higher among females) in Bulgaria, Lithuania, Portugal, Sweden, Switzerland, Montenegro, Albania, and Turkey. The indicator of children living in households with very low work intensity exhibited a significant difference in terms of gender (being mostly higher among males) in Bulgaria, Lithuania, Malta, the Netherlands, Finland, Sweden, Switzerland, and Albania. The indicator of severe social and material deprivation exhibited a significant difference in terms of gender (being mostly higher among females) in Bulgaria, Denmark, Estonia, Lithuania, Montenegro, Albania, and Turkey. Serbia did not show a significant gender difference, but the male gender had higher values than the female for all indicators. **Conclusions**: Child poverty is a multifaceted issue that affects various aspects of children’s lives, including health, education, and social inclusion. Addressing this issue requires a comprehensive approach that includes social protection, access to quality services, and efforts to combat discrimination.

## 1. Introduction

Child poverty is a critical issue that affects millions of children worldwide and represents a global issue. It encompasses not only the lack of financial resources but also limited access to essential services such as healthcare, education, and social participation [1]. It occurs in low-, middle-, and high-income countries to varying degrees, and nearly one in four children in Europe are at risk of poverty and social exclusion [2].

The risk of child poverty refers to the likelihood that children live in households with income levels insufficient to meet basic needs, such as adequate food, clothing, housing, and access to essential services [3]. In Serbia, as in many countries, children are more likely to experience poverty than adults [4]. The factors contributing to this risk include low parental incomes, unemployment, single-parent households, and a lack of access to quality education and healthcare services [5]. In most countries, especially ones with more pronounced traditional gender roles, females are at a higher risk of poverty and social exclusion. In many Southern and Eastern European countries, the main role of women is to primarily be responsible for childcare and domestic work. Women are influenced in this way from a young age, constructing their role and personal development, and thus impacting their access to education, limiting their career pathways, and decreasing their independence [6].

It is important to deal with this subject for a few main reasons: it influences human rights, as every child has a right to an adequate standard of living; it supports equal opportunities for children by promoting social cohesion and reducing disparities in society; and finally it influences economic growth because a healthier and better-educated society is essential for economic development [7,8]. Children can also experience inequality among genders in their education. The development of society is very dynamic, and a lot of patterns of behavior and opportunities were different at times when economic crises emerged from every corner, pushing education to the sidelines [9]. The past has led to the creation of different values, thus influencing gender inequality, mainly by placing females in an unfavorable position, but males also. Even now, a lot of rural places in a given country, either less or more developed, have higher rates of males with lower educational attainment and higher social exclusion rates due to their involvement in more physical jobs [10].

There are several impactful effects of child poverty on a child’s life. Children living in poverty are more likely to experience poor health outcomes. They often have limited access to nutritious food, clean water, and healthcare services, leading to higher rates of malnutrition, chronic illnesses, and developmental delays [11]. Poverty affects children’s ability to succeed in education. A lack of resources can lead to poor school attendance, lower academic achievement, and higher dropout rates [12]. The stress associated with living in poverty can lead to emotional and psychological issues in children, including anxiety, depression, and behavioral problems [13]. Gender differences in Europe have shifted over the past few decades. While males once outperformed females, today females tend to perform better in most academic areas and also in school attainment rates [14]. According to Eurostat data from 2023, more females graduated from tertiary education than males (54% vs. 46%), while school leaving was more common among males (11% vs. 7%) [15]. There are several reasons suggested to explain this difference, with the main focus being on the sociocultural norms. Girls are often encouraged to be more communicative and compliant, and boys to be more socialized, active, and physical.

Children in poverty are often excluded from social activities and opportunities, leading to feelings of isolation and stigmatization. This exclusion can affect their social development and sense of belonging [16]. Social exclusion refers to the processes through which individuals or groups are systematically disadvantaged and marginalized. Children in poverty are at a higher risk of social exclusion due to inadequate access to education, healthcare, and social services; marginalization based on ethnicity, disability, or other characteristics; and negative societal attitudes toward poverty—i.e., stigmatization [17,18,19,20]. Lower-income countries have more boys leaving school to work, while in traditional communities, girls’ education may be undervalued. Many children from national minorities in European countries experience a higher rate of social exclusion and poverty, as they are forced to attend lower-quality schools or are sometimes wrongly placed in special education [21]. Stigma leads to lower expectations from teachers, which affects these children’s performance and motivation, and leads to early school leaving, especially among Roma children [22]. This has been the situation for a very long time in many Balkan countries, including Serbia. In Germany and the Netherlands, this is the situation with these countries’ Turkish minorities, and in France and Belgium, with North Africans.

Healthcare plays a vital role in addressing the impacts of child poverty by ensuring that all children have access to essential health services, identifying and addressing health issues early to prevent long-term consequences, and providing support to families to improve overall well-being. Healthcare systems can collaborate with other sectors, such as education and social services, to provide comprehensive support to children and families [23,24]. Although universal health coverage propagates equal health to all, this situation is still abstract in many countries worldwide. Some cultures prioritize boys’ health needs over those of girls, especially in patriarchal and traditional families, and in some marginalized ethnic groups girls receive less investment in their health. Sexual education is very limited in some countries, leaving both genders with much less information than necessary, which leads to poorer health literacy and health outcomes, especially in adolescents, such as unwanted pregnancies and higher rates of sexually transmitted diseases. Similarly, but in the area of mental health, boys tend to seek help less than girls due to sociocultural upbringing [25].

Serbia is an EU enlargement candidate country, considered to be an upper-middle-income country by the World Bank [26]. It has a post-socialist transitioning economy, which is still recovering from the 1990s conflicts and economic sanctions. Compared to EU countries, Serbia ranks in the lower third in income and development but performs better than some Western Balkan countries, and worse than most EU countries. It has a very traditional position regarding gender roles, which are slowly changing, but are still very present. It has an overall poverty rate of 21% of the population according to Eurostat, compared with an average of 17% in Europe, while every third child is considered to be at risk of poverty. The health system underperforms even while free and low-cost public health services are available, due to being overburdened and underfunded. The position of Serbia is very similar to many of its neighboring countries due to their similar historical and developmental pathways [27]. This article focuses on exploring gender inequality within the risk of child poverty and social exclusion across Europe while comparing the position of Serbia to other countries with different levels of development. We wanted to emphasize its significance, the multifaceted impacts it has on children, its connection to social exclusion, as well as the crucial role of healthcare in addressing these challenges.

## 2. Materials and Methods

This is an epidemiological, retrospective, and descriptive study based on data on the national populations of the European countries within our research interest. The data analyzed in this study were taken from publicly available datasets from Eurostat [28]. The indicators of interest were those considering the social inclusion of children in the time period between 2014 and 2023 for all European countries available in the datasets (32 to 36, based on the availability of data).

The following indicators were analyzed: the rate of children (aged less than 18) at risk of poverty or social exclusion, the at-risk-of-poverty rate for children (aged less than 18) by gender, children (aged less than 18) living in households with very low work intensity by gender, and the severe material and social deprivation rate for children by gender.

The indicator at risk of poverty or social exclusion corresponds to the sum of persons who are at risk of poverty or are severely materially or socially deprived, or are living in households with very low work intensity. Persons are only counted once, even if they are present across several sub-indicators. Those at risk of poverty are persons with an equalized disposable income below the risk-of-poverty threshold, which is set at 60% of the national median equalized disposable income (after social transfers). Severely materially or socially deprived persons have living conditions severely constrained by a lack of resources, they experience at least 7 out of 13 of the following deprivation items: cannot afford (1) to pay rent or utility bills; (2) to keep their home adequately warm; (3) to face unexpected expenses; (4) to eat meat, fish, or a protein equivalent every second day; (5) a week-long holiday away from home; (6) to have access to a car/van for personal use; (7) to replace worn out furniture; (8) to replace worn-out clothes with some new ones; (9) to have two pairs of properly fitting shoes; (10) to spend a small amount of money each week on him/herself (“pocket money”); (11) to engage in regular leisure activities; (12) to get together with friends/family for a drink/meal at least once a month; and (13) to have an internet connection. People living in households with very low work intensity are those aged 0–64 living in households where the adults (aged 18–64) worked 20% or less of their total work potential during the past year. The indicator is based on the European Union Statistics on Income and Living Conditions—EU-SILC (statistics on income, social inclusion, and living conditions).

The investigation used data from a publicly available database, so ethical consideration was not necessary, nor was consent from the participants, because the data were anonymous and collected by national authorities according to the International Ethical Guidelines for Biomedical Research Involving Humans, as well as WHO Good Clinical Practice.

The data was analyzed using descriptive statistics, min, max, mean, and standard deviation. Linear regression was analyzed to assess the type of progress during the observed period, using the formula Y = A+ BX. The dependent variable is Y, the independent variable is X, B is the slope of the line, and A is the point where Y intercepts the line. Regression also gives an R-squared value; the values range from 0 to 1. The confidence interval for prediction was 95%. Linear regression shows if the values follow a positive or negative type of growth over time, or whether they are stagnating. The normality of variables was assessed. Pearson correlation was performed to assess the connection between male and female values for the indicators of interest during the observed period, to determine if there is a positive or negative correlation between them. Afterward, the *t*-test was used to assess the existence of a statistical difference between genders, where a value of *p* below 0.05 was taken as statistically significant. Analyses were conducted using the IBM SPSS software package Version 26.0. (The Statistical Package for the Social Sciences software) (Version 26.0, SPSS Inc., Chicago, IL, USA).

## 3. Results

It is noted that among the 32 European countries, most experienced a decrease in the rate of children (aged less than 18) at risk of poverty or social exclusion throughout the observed decade (2015–2023). To be exact, 24 countries experienced a decrease, where the most prominent decreases were observed in Cyprus (y = −0.7767x + 24.061; R^2^ = 0.9591), Lithuania (y = −1.8317x + 36.125; R^2^ = 0.9317), and Greece (y = −1.3x + 39.356; R^2^ = 0.921). In contrast, eight countries experienced increases, including Germany, Spain, France, Luxemburg, Austria, Sweden, Norway, and Turkey, where the most prominent increases were in Germany (y = 0.7467x + 17.456; R^2^ = 0.6684), France (y = 0.405x + 22.153; R^2^ = 0.4608), and Turkey (y = 0.8483x + 36.969; R^2^ = 0.4469). The results of the descriptive statistics and linear regression are presented in Table 1. Serbia is among the countries where a decrease is noticeable, while the percentage between the first and last studied year dropped by roughly 15%, indicating progress regarding the observed indicator.

**Figure 1:**
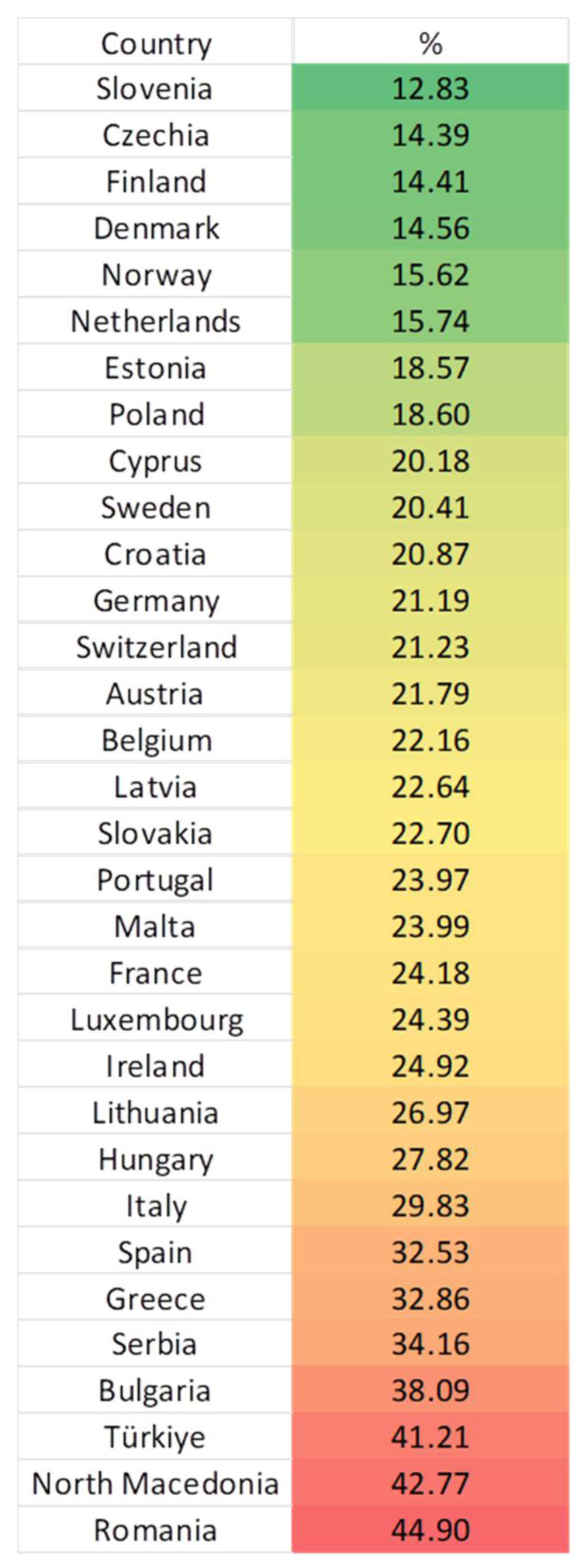
A heat map representing mean % values (2015–2023) for the indicator children (aged less than 18) at risk of poverty or social exclusion, across Europe (lighter colors represent lesser risk).

The country with the lowest percentage of children at risk of poverty was Slovenia (12.83%), while Romania was the country with the highest percentage (44.9%). Considering Serbia, in comparison to the rest of Europe, it was ranked as the fifth country with the highest percentage of children at risk of poverty or social exclusion (Figure 1).

**Figure 2:**
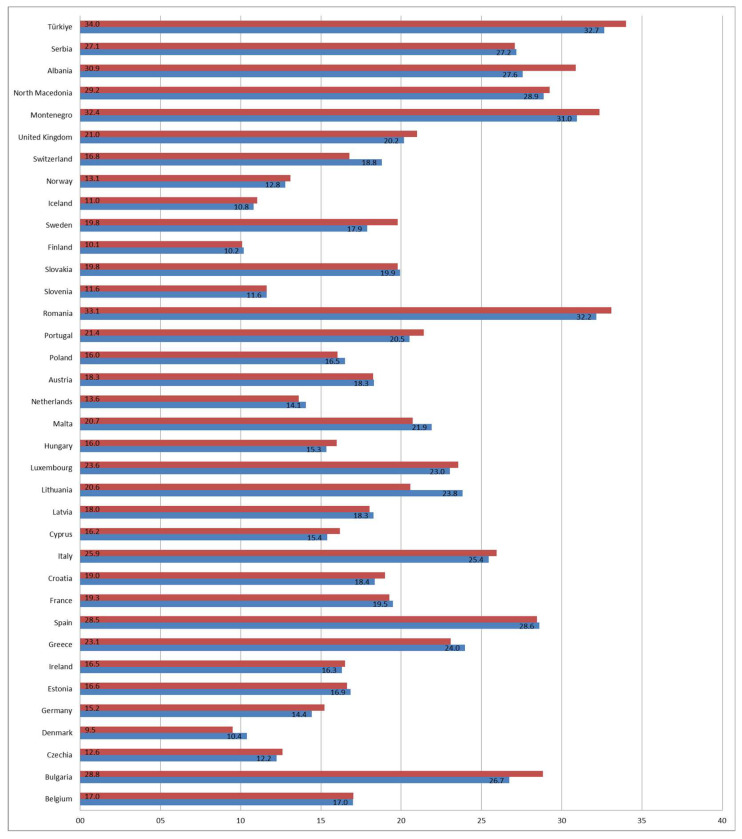
Country-specific mean % values for the at-risk-of-poverty rate for children in the observed period 2015–2023 by gender (blue represents males and red females).

Correlation was examined between males and females for the at-risk-of-poverty rate for children (aged less than 18) in the observed decade to determine the existence of a linear correlation. Most countries showed a significant positive correlation for at risk of poverty, 22 out of 36 countries, and Serbia was among them, while 14 showed no correlation. The *t*-test found a significant difference between males and females in nine observed countries, while in three countries the risk of poverty was higher for males (Denmark, Lithuania, and Switzerland), and in six for females (Bulgaria, Portugal, Sweden, Montenegro, Albania, and Turkey) (Table 2). The mean value for males was highest in Turkey (32.7%) and lowest in Finland (10.2%), and for females, the highest was also Turkey (34.0%) while the lowest was in Denmark (9.5%). Serbia had a mean overall value for both genders of roughly 27%, indicating that almost every third male and female child is at risk of poverty (Figure 2).

**Figure 3:**
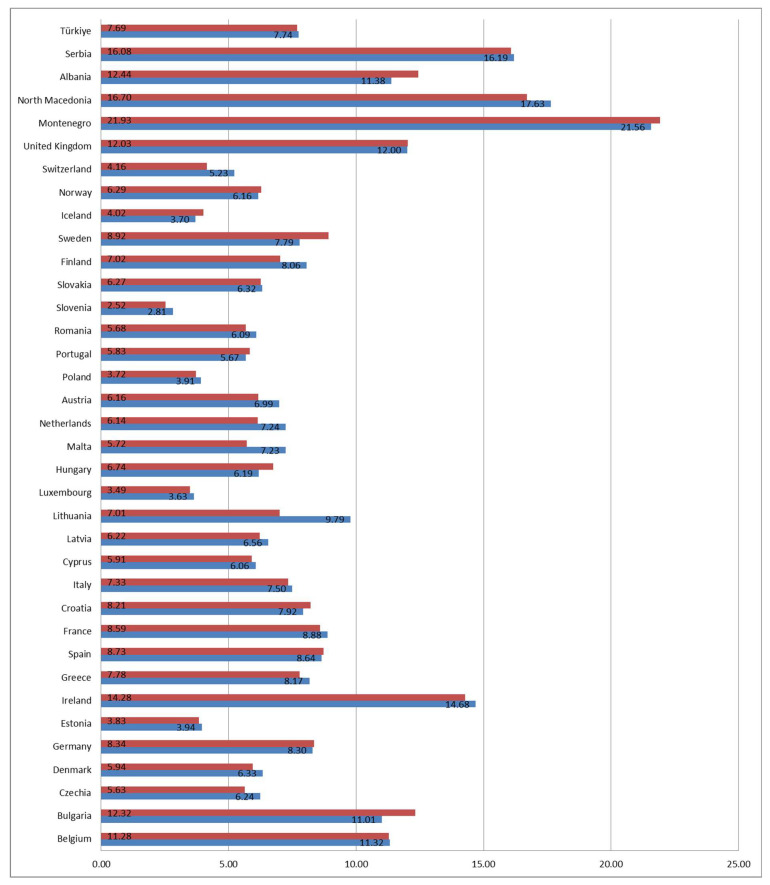
Country-specific mean % values of the indicator children living in households with very low work intensity in the observed period 2015–2023 by gender (blue represents males and red females).

Correlations between male and female children for the indicator children (aged less than 18) living in households with very low work intensity were also assessed in our investigation to establish the existence of a linear correlation and the results show that it existed in most countries—24, with Serbia being one of them. Twelve countries showed no significant correlation. The *t*-test found significant differences between males and females in eight of the observed countries, where in six countries the indicator was higher for males (Lithuania, Malta, Netherlands, Finland, Switzerland, and North Macedonia), and in two countries it was higher for females (Bulgaria and Sweden) (Table 2). The mean value for males was highest in Montenegro (21.6%), followed by North Macedonia and Serbia; the same as for females, with Montenegro having the highest percentage (21.9%) (Figure 3).

**Figure 4:**
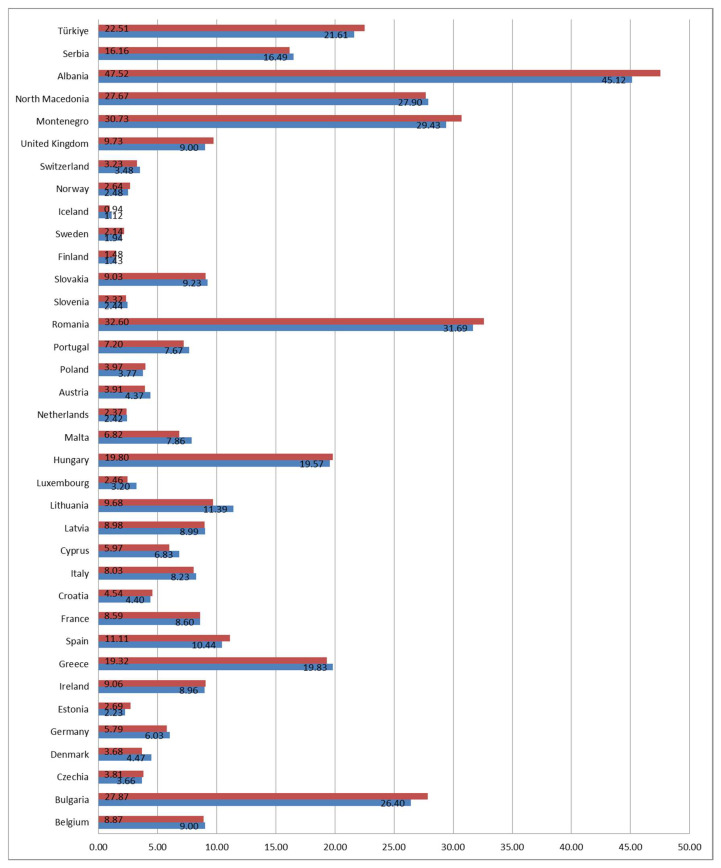
Country-specific mean % values of the indicator severe material and social deprivation rate for children aged less than 18 in the observed period 2015–2023 by gender (blue represents males and red females).

Finally, the correlation between males and females for the indicator severe material and social deprivation rate for children in the observed decade was examined to determine the existence of a linear correlation. Most countries showed a significant positive correlation for the severe material and social deprivation rate by gender, and out of 36 countries, 26 showed a significant correlation, while 10 of them showed no correlation. The *t*-test found significant differences between males and females in seven of the observed countries, where the indicator was higher in males in two countries (Denmark and Lithuania) and in five countries it was higher for females (Bulgaria, Estonia, Montenegro, Albania, and Turkey) (Table 2). The mean value for males and females was highest in Albania (45.1% and 47.5%) and lowest in Iceland and Finland (1.4% and 1.5%), which can be seen in Figure 4.

## 4. Discussion

The study by Schenck-Fontaine highlights that many children experience material hardship even if their families earn above the federal poverty line, challenging the traditional view of economic vulnerability. They described four types of material hardship: basic expense hardship, food insecurity, housing hardship, and medical hardship. Due to different types of material hardship, we have to be knowledgeable about the different ways it impacts children, either in the shorter or the longer term, which has important implications for both research and policy in order to better support vulnerable children [29].

As of 2023, nearly 20 million children in the European Union (EU), representing 24.8% of all children under 18 years of age, were at risk of poverty or social exclusion, a figure that remained relatively stable compared to 2022 [30]. The EU average stood at 24.8% in 2023, with the highest rates in Romania, Spain, and Bulgaria, while the lowest were in Slovenia, Finland, and the Netherlands. Even high-income countries like France (26.6%) and Luxembourg (26.1%) report concerning levels. In EU enlargement countries, child poverty remains high, with 27.1% in Serbia and 30.2% in Moldova [31]. And, as previously stated, children compared to adults were at a higher risk (24.8% vs. 20.6%). In our results, the European country with the highest rates of poverty or social exclusion was Romania, and Slovenia had the lowest rates. Both of these countries showed decreasing trends over the observed period, and Germany showed the most prominent increasing trend, with a percentage increase of 4.6. Gender inequality is very present in Turkey, and even women’s non-governmental organizations (NGOs), often assumed to be feminist, can harbor gender-inequitable attitudes. Influenced by education, class, and political beliefs, these organizations may unintentionally reproduce patriarchal norms [32,33]. Our study showed that Turkey has the highest percentages of females and males at risk of poverty and social exclusion, with females having the higher percentage, which confirms statements from the previous study.

In 2022, the EU average for children living in severe material and social deprivation was 8.4%. Romania, Bulgaria, and Greece had the highest rates, while Austria (2.2%), Slovenia, and Finland reported the lowest [34]. Among the countries with the highest percentages, it was notable that gender inequality was present more among females. Gender and race were brought up as the factors that must be addressed due to their influence on academic performance, as they have impactful effects on children [35]. Although the difference among genders in severe material and social deprivation is not so deeply pronounced in childhood, in later life it is proven that females are more affected than males [36]. We also confirmed that in more countries where a significant difference between genders was found, females had higher percentages, especially in Albania and Romania. The COVID-19 pandemic reversed the declining trend in child poverty. In 2020, the number of children experiencing severe material deprivation increased by 0.9 million in the EU, marking a 19% rise compared to 2019 [37,38]. In our results, nine of the observed countries experienced an increase in the indicator children at risk of poverty and social exclusion. One study in North Macedonia used microsimulation models and estimated the change in poverty rates during the COVID pandemic, which increased by nearly 5%, resulting in an estimated additional 19,000 children being affected by poverty [39].

The EU has allocated substantial funds to address child poverty via the European Social Fund+ (ESF+) and the European Regional Development Fund (ERDF), and by promoting social inclusion and combating poverty, notably by supporting investment in social infrastructure and access to quality services [40].

The study by Benedetti et al. offers a comprehensive assessment of child poverty rates across 33 European nations, utilizing Eurostat data. It identifies higher child poverty rates in Southern Europe and the UK, while countries like the Czech Republic, Malta, and Poland report lower rates [41]. The research emphasizes the importance of parental employment and the efficiency of child-related income transfers in combating child poverty. Despite the results of their research not considering gender differences, our research found gender differences in Montenegro, Albania, and Bulgaria for females.

An article published by the International Monetary Fund examined the significant increase in child poverty during the COVID-19 pandemic, noting a 19% rise in 2020. It advocates for policies that enhance parental labor income and increased public spending on family and child support, alongside investments in education, childcare, health, and housing, to mitigate long-term impacts [34]. During the pandemic, many families faced the problem of limitations regarding job opportunities, and issues with retaining steady work and incomes. Our results also examined the aspect of children living in very low work intensity households and gender differences. Interestingly, these differences were found in many Northern European countries for males, and some Eastern European countries for females. Most Northern European countries are among the group of developed countries, with higher incomes and where gender equality is very high. Thus, the structure of families is very different in comparison to other European countries. Cohabiting couples with children, single parents, and divorce rates are much higher than in other countries; also, boys may be more present in households with higher vulnerability [42].

Robust social spending and supportive family structures are important in terms of reducing child deprivation, and Pérez-Corral et al. imply that the role of family policies and social protection systems in influencing child poverty rates is crucial. Greater social spending on sickness and disability benefits can reduce the risk of child deprivation, particularly for single-parent families [43].

The study by Bouillet et al. examined the accessibility and quality of Early Childhood Education and Care (ECAS) in Croatia, focusing on children at risk of social exclusion. The study identified poverty and ethnic minority status as significant barriers to ECEC participation [44]. We identified Croatia as a country with a steady, decreasing trend of the indicator at risk of poverty and social exclusion with a lower risk in comparison to other countries, and without significant gender inequality.

New ways of dealing with social exclusion among adolescents by using serious games were identified in the review article and highlighted how digital interventions can address issues like anxiety, mental health problems, and social isolation, which have been exacerbated by the COVID-19 pandemic [45].

Child poverty in Europe is closely linked to family structure. Large families and single-parent households face a higher risk of poverty. However, European countries vary in how effectively they reduce child poverty, with some doing better at supporting large families than others. These differences highlight the need for tailored social policies across the region [46].

The recommendations issued by the Council of Europe and the European Commission convey a clear directive to ensure the fulfillment and safeguarding of children’s rights; it is essential to provide adequate support for families. These entities further propose that a highly effective means of achieving this objective is through the implementation of redistributive policies and the provision of inclusive, progressively accessible services designed to guarantee children an appropriate standard of living. However, both organizations lack the authority to impose national policies, as such decisions are made independently by each member state. Regrettably, the adoption of these policy recommendations has been limited to date. Consequently, it is crucial to emphasize the significance of the recommendations put forward by the Council of Europe and the European Commission, urging national policymakers to incorporate these proposals into their domestic agendas [40].

Welfare system reforms in most European countries have been characterized by incremental change and continuity, rather than radical transformation. These reforms often build upon existing structures and long-standing traditions, which reflect historical practices of social negotiation. This demonstrates a strong path dependency, where past policies shape present decisions. The only significant exception is Poland, which, due to its post-socialist transition, was more open to implementing deep and structural reforms in its welfare system. Overall, the evolution of welfare systems in Europe shows a preference for adaptation over disruption [47]. While stability and continuity can protect social cohesion, excessive caution may prevent welfare systems from evolving enough to meet the complex, fast-changing needs of modern societies (e.g., rising inequality). This can leave vulnerable groups under-supported and can widen social exclusion.

The limitations of our research are that we focused only on exploring the gender inequalities among European countries for the indicators children at risk of poverty, children living in households with very low work intensity, and the severe material and social deprivation rate for children, but did not focus on indicators that may influence these differences. Indicators such as family education status, the economic status of the parents, household type, ethnic minority status, and the age of the children can influence the indicators of poverty and social exclusion. This research presents a good base from which future articles can focus on those factors as well.

## 5. Conclusions

The indicator at risk of poverty and social exclusion showed a decreasing trend in the majority of the countries; but still, seven countries showed an increase during the observation period of one decade. The highest percentage was in Romania and the lowest in Slovenia. The indicator at risk of poverty exhibited significant differences in terms of gender (being mostly higher among females) in Bulgaria, Lithuania, Portugal, Sweden, Switzerland, Montenegro, Albania, and Turkey. The indicator of children living in households with very low work intensity exhibited significant differences in terms of gender (being mostly higher among males) in Bulgaria, Lithuania, Malta, the Netherlands, Finland, Sweden, Switzerland, and Albania. The indicator of severe social and material deprivation exhibited significant differences in terms of gender (being mostly higher among females) in Bulgaria, Denmark, Estonia, Lithuania, Montenegro, Albania, and Turkey. Serbia did not show a significant gender difference, but the male gender had higher values than females for all indicators.

Child poverty is a multifaceted issue that affects various aspects of children’s lives, including health, education, and social inclusion. Vulnerable groups such as children from migrant backgrounds, single-parent or large families, children with disabilities, and ethnic minorities are disproportionately affected. Gender inequality is still very present due to cultural differences and social norms, regardless of the level of a country’s development. Addressing this issue requires a comprehensive approach that includes social protection, access to quality services, and efforts to combat discrimination. By investing in children and ensuring they have the resources and support they need, societies can break the cycle of poverty and build a more equitable and prosperous future. Gender-sensitive interventions and policy design need to be implemented and promoted to a greater extent in European countries.

## Figures and Tables

**Table 1:** Children (aged less than 18) at risk of poverty or social exclusion; descriptive statistics and linear regression with additional values for the first and last observed year.

	2015	2023	Min	Max	Median	SD	Linear Regression
Belgium	24.1	19	19.00	24.20	22.16	1.99	y = −0.6833x + 25.572; R^2^ = 0.8882
Bulgaria	47.5	33.9	33.00	47.50	38.09	5.98	y = −1.8567x + 47.372; R^2^ = 0.7232
Czechia	17.8	15	12.90	17.80	14.39	1.72	y = −0.3583x + 16.181; R^2^ = 0.3266
Denmark	16.2	15.3	13.40	16.20	14.56	0.96	y = −0.14x + 15.256; R^2^ = 0.1597
Germany	19.3	23.9	17.50	24.40	21.19	2.50	y = 0.7467x + 17.456; R^2^ = 0.6684
Estonia	22.2	18.3	16.60	22.20	18.57	1.77	y = −0.455x + 20.842; R^2^ = 0.4981
Ireland	29.2	24.3	22.30	29.20	24.92	2.20	y = −0.6733x + 28.289; R^2^ = 0.7026
Greece	37.7	28.1	28.10	37.70	32.86	3.71	y = −1.3x + 39.356; R^2^ = 0.921
Spain	34.0	34.5	30.50	34.50	32.53	1.41	y = 0.0267x + 32.4; R^2^ = 0.0027
France	22.4	26.6	22.40	27.10	24.18	1.63	y = 0.405x + 22.153; R^2^ = 0.4608
Croatia	25.2	17.3	17.30	25.20	20.87	3.18	y = −1.1017x + 26.375; R^2^ = 0.8995
Italy	34.1	27.1	27.10	34.10	29.83	2.37	y = −0.725x + 33.458; R^2^ = 0.7005
Cyprus	22.9	16.7	16.70	23.20	20.18	2.17	y = −0.7767x + 24.061; R^2^ = 0.9591
Latvia	30.7	20.3	18.70	30.70	22.64	3.97	y = −1.2267x + 28.778; R^2^ = 0.7152
Lithuania	34.8	21.7	21.60	34.80	26.97	5.20	y = −1.8317x + 36.125; R^2^ = 0.9317
Luxembourg	23.3	26.1	21.50	29.40	24.39	2.36	y = 0.5533x + 21.622; R^2^ = 0.4124
Hungary	40.3	24.4	18.10	40.30	27.82	7.78	y = −2.5033x + 40.339; R^2^ = 0.7766
Malta	27.8	25.2	22.30	27.80	23.99	1.62	y = −0.2333x + 25.156; R^2^ = 0.1552
Netherlands	17.1	15.9	13.90	17.20	15.74	1.06	y = −0.2833x + 17.161; R^2^ = 0.5315
Austria	22.2	22.7	20.10	22.80	21.79	0.96	y = 0.0967x + 21.306; R^2^ = 0.0753
Poland	26.8	16.9	16.10	26.80	18.60	3.84	y = −1.0567x + 23.883; R^2^ = 0.5692
Portugal	31.2	22.6	20.70	31.20	23.97	3.35	y = −0.9783x + 28.858; R^2^ = 0.6389
Romania	53.4	39.0	39.00	56.00	44.90	6.32	y = −1.9833x + 54.817; R^2^ = 0.7385
Slovenia	16.6	10.7	10.30	16.60	12.83	2.27	y = −0.7733x + 16.7; R^2^ = 0.8735
Slovakia	24.7	25.3	18.40	25.30	22.70	2.47	y = −0.1317x + 23.358; R^2^ = 0.0213
Finland	14.5	13.8	13.20	15.90	14.41	0.80	y = −0.09x + 14.861; R^2^ = 0.0948
Sweden	19.7	21.6	19.40	23.00	20.41	1.17	y = 0.1417x + 19.703; R^2^ = 0.1098
Norway	13.6	15.0	13.60	17.20	15.62	1.17	y = 0.2333x + 14.456; R^2^ = 0.2987
Switzerland	20.6	22.4	19.30	22.80	21.23	1.29	y = −0.0133x + 21.3; R^2^ = 0.0008
North Macedonia	47.0	39.7	38.40	47.00	42.77	3.30	y = −6.905x + 63.036; R^2^ = 0.7706
Serbia	42.4	27.1	27.10	42.40	34.16	5.69	y = −2.0869x + 43.554; R^2^ = 0.8084
Turkey	35.5	40.3	35.50	45.20	41.21	3.48	y = 0.8483x + 36.969; R^2^ = 0.4469

**Table 2:** Pearson correlation and *t*-test between genders for the indicators of interest, for children aged less than 18 years.

	At-Risk-of-Poverty Rate for Children		*t*-Test	Children Living in Households with Very Low Work Intensity		*t*-Test	Severe Material and Social Deprivation Rate for Children		*t*-Test
	Pearson Correlation	Sig.	*p*-value	Pearson Correlation	Sig.	*p*-value	Pearson Correlation	Sig.	*p*-value
Belgium	0.671	0.034	0.988	0.738	0.023	0.931	0.600	0.088	0.757
Bulgaria	0.824	0.003	0.003	0.951	0.000	0.000	0.996	0.000	0.002
Czechia	0.652	0.041	0.428	0.839	0.005	0.109	0.859	0.003	0.646
Denmark	−0.330	0.352	0.077	0.147	0.706	0.513	0.766	0.016	0.033
Germany	0.470	0.170	0.102	0.969	0.000	0.847	0.978	0.000	0.167
Estonia	0.698	0.025	0.695	0.200	0.606	0.745	0.827	0.006	0.010
Ireland	0.553	0.097	0.713	0.965	0.000	0.240	0.689	0.040	0.873
Greece	0.804	0.005	0.073	0.986	0.000	0.055	0.972	0.000	0.159
Spain	0.596	0.069	0.751	0.917	0.000	0.759	0.322	0.398	0.082
France	0.513	0.129	0.648	0.928	0.000	0.215	0.506	0.165	0.977
Croatia	0.810	0.005	0.203	0.972	0.000	0.584	0.952	0.000	0.587
Italy	0.152	0.674	0.307	0.638	0.043	0.594	0.990	0.000	0.307
Cyprus	0.582	0.078	0.156	0.933	0.000	0.718	0.900	0.001	0.058
Latvia	0.849	0.002	0.674	0.526	0.146	0.181	0.983	0.000	0.970
Lithuania	0.850	0.002	0.002	0.042	0.915	0.011	0.924	0.000	0.036
Luxembourg	0.529	0.116	0.563	0.832	0.005	0.569	0.065	0.867	0.185
Hungary	0.983	0.000	0.114	0.920	0.000	0.197	0.979	0.000	0.725
Malta	0.090	0.804	0.240	0.847	0.004	0.005	0.637	0.065	0.092
Netherlands	0.033	0.929	0.244	0.425	0.254	0.002	−0.062	0.874	0.842
Austria	0.798	0.006	0.890	0.607	0.083	0.053	0.533	0.140	0.303
Poland	0.983	0.000	0.199	0.890	0.001	0.205	0.976	0.000	0.487
Portugal	0.947	0.000	0.021	0.849	0.001	0.522	0.962	0.000	0.215
Romania	0.823	0.003	0.274	0.853	0.003	0.190	0.974	0.000	0.193
Slovenia	0.821	0.004	0.980	0.623	0.073	0.217	0.933	0.000	0.496
Slovakia	0.518	0.125	0.824	0.584	0.098	0.934	0.875	0.002	0.640
Finland	0.034	0.926	0.831	0.644	0.061	0.010	0.665	0.051	0.790
Sweden	0.431	0.214	0.003	0.533	0.140	0.002	0.787	0.012	0.387
Iceland	0.991	0.000	0.627	0.949	0.000	0.468	0.922	0.000	0.387
Norway	0.859	0.001	0.249	0.540	0.134	0.704	0.592	0.093	0.672
Switzerland	0.669	0.034	0.003	0.364	0.335	0.029	0.723	0.028	0.402
United K	0.997	0.000	0.236	0.994	0.000	0.963	0.997	0.000	0.113
Montenegro	0.988	0.000	0.035	0.990	0.000	0.432	0.992	0.000	0.046
N. Macedonia	0.995	0.000	0.595	0.990	0.000	0.201	0.999	0.000	0.555
Albania	0.999	0.000	0.019	0.998	0.000	0.035	1.000	0.000	0.019
Serbia	0.994	0.000	0.800	0.963	0.000	0.878	0.978	0.001	0.709
Turkey	0.860	0.001	0.000	0.960	0.000	0.578	0.999	0.000	0.013

## Data Availability

Data used for this research can be found at the following link https://ec.europa.eu/eurostat/data/database (accessed on 25 May 2025).
